# The Relationship Between Dysphagia and Pneumonia in Acute Stroke Patients: A Systematic Review and Meta-Analysis

**DOI:** 10.3389/fneur.2022.834240

**Published:** 2022-03-17

**Authors:** Min Cheol Chang, Yoo Jin Choo, Kyung Cheon Seo, Seoyon Yang

**Affiliations:** ^1^Department of Physical Medicine and Rehabilitation, College of Medicine, Yeungnam University, Daegu, South Korea; ^2^Department of Rehabilitation Medicine, Asan Medical Center, University of Ulsan College of Medicine, Ulsan, South Korea; ^3^Department of Rehabilitation Medicine, Ewha Womans University Seoul Hospital, School of Medicine, Ewha Womans University, Seoul, South Korea

**Keywords:** pneumonia, stroke, mortality, meta-analysis, dysphagia

## Abstract

**Background:**

Dysphagia is a common complication after stroke and is associated with the development of pneumonia. This study aimed to summarize the relationship between dysphagia and pneumonia in post-stroke patients.

**Materials and Methods:**

Articles published up to November 2021 were searched in the PubMed, Embase, Cochrane library, and Scopus databases. Studies that investigated the development of pneumonia in acute stroke patients with and without dysphagia were included. The methodological quality of individual studies was evaluated using the Risk Of Bias In Non-randomized Studies-of Interventions tool, and publication bias was evaluated using a funnel plot and Egger's test.

**Results:**

Of 5,314 studies, five studies were included in the meta-analysis. The results revealed that the incidence of pneumonia was significantly higher in the dysphagia group than in the non-dysphagia group (OR 9.60; 95% CI 5.75–16.04; *p* < 0.0001; *I*^2^ = 78%). There was no significant difference in the mortality rate between the two groups (OR 5.64; 95% CI 0.83–38.18; *p* = 0.08; *I*^2^ = 99%).

**Conclusion:**

Dysphagia is a significant risk factor for pneumonia after stroke. The early diagnosis and treatment of dysphagia in stroke patients are important to prevent stroke-associated pneumonia.

## Introduction

Stroke is one of the leading causes of disability and mortality worldwide (1, 2). The number of stroke patients is gradually increasing around the world, leading to increases in healthcare costs (3). Patients with stroke have various clinical conditions, including hemiparesis, loss of dexterity, functional limitations, gait disturbance, cognitive impairment, dysarthria, spasticity, neglect, and dysphagia (4).

Dysphagia is a symptom characterized by difficulties in the passage of food or liquid from the mouth through the pharynx, esophagus, and stomach (5). The difficulty in swallowing can occur in any of the four phases (i.e., oral preparatory, oral, pharyngeal, and esophageal phases) (6). Dysphagia occurs in ~3% of the general population, frequently affecting the elderly and patients with cerebrovascular accidents (7). The prevalence of dysphagia after stroke has been reported to be around 30–65% (2, 8). Post-stroke dysphagia may show improvements during the first week after stroke; however, it may also persist as a chronic condition with many complications. The presence of dysphagia in stroke patients may cause poor dietary intake, dehydration, malnutrition, and pulmonary complications, which can lead to poor prognosis (9).

Stroke-associated pneumonia is one of the most common post-stroke infections with a prevalence of 14.3% (10). It affects clinical outcomes and is associated with an increased risk of prolonged hospital stay and poor recovery (11). Pneumonia is the leading cause of death during the acute phase of stroke, with a 30-day mortality rate of 30% (12). Dysphagia is one of the most significant risk factors in the development of pneumonia (2). Aspiration pneumonia is defined as pneumonia with preexisting risk factors accompanying demonstrated or suspected aspiration (13). The presence of oropharyngeal aspiration is associated with various clinical symptoms, including choking, coughing, or a wet-sounding voice during or after eating (14). In addition, it may occur without cough or airway protective responses, making diagnosis more difficult (14). Patients with dysphagia have a higher risk of aspiration, leading to an increased risk of acquiring pneumonia. Previous animal models and human clinical studies have shown that the primary cause of aspiration pneumonia is silent aspiration (15). It has been reported that stroke patients with dysphagia have a 3- to 11-fold increased risk of acquiring pneumonia, and the prevalence of pneumonia is higher among patients with dysphagia compared with those without dysphagia (8).

Considering that both dysphagia and aspiration are highly prevalent in stroke patients, clarifying the relationship between dysphagia and pneumonia can contribute to the early detection and better management of dysphagia in stroke patients. Investigating the risk factors of pneumonia can help reduce post-stroke infections and associated complications. The objective of this systematic review and meta-analysis was to investigate and summarize the effect of dysphagia on pneumonia and mortality in patients with stroke.

## Methods

A systematic review of studies related to post-stroke dysphagia and complications (pneumonia and mortality) in patients with stroke was conducted.

### Search Strategy

This meta-analysis was performed according to the Preferred Reporting Items for Systematic Reviews and Meta-Analyses (PRISMA) guidelines (16). The protocol of this meta-analysis was registered on INPALSY (International Platform of Registered Systematic Review and Meta-analysis Protocols) with a registration number of INPALSY2021110108. Relevant articles were systematically searched using the PubMed, Embase, Cochrane library, and Scopus databases up to November 2021. The following keywords were used in the search: “deglutition disorders,” “deglutition,” “dysphagia,” “swallowing,” “deglutition pneumonia,” and “aspiration pneumonia.” Used search terms and strategies are presented in Supplementary Appendix 1.

### Study Selection

The eligibility criteria for this meta-analysis were based on the Population. Intervention, Comparison, and Outcome (PICO) framework (17). Using the PICO strategy, patients with acute stroke were classified as the population, and patients with and without dysphagia were classified as the intervention and comparison groups, respectively. The main outcomes of interest were the incidence of pneumonia and number of deaths attributed to post-stroke dysphagia. The following inclusion criteria were used for the selection of articles: (1) acute stroke patients were recruited; (2) dysphagia was diagnosed in the intervention group (dysphagia group) and not diagnosed in the comparison group (control group); (3) development of pneumonia or number of deaths were evaluated in both groups. The exclusion criteria were as follows: (1) studies not related to dysphagia, pneumonia, or mortality; (2) reviews, case reports, commentaries, letters, and animal studies; (3) study outcomes that were not reported or insufficient. Two independent reviewers excluded articles after reading the titles and abstracts (KCS and SYY), and full-text assessments were conducted to reject those not fulfilling the inclusion criteria. The reviewers attempted to resolve any disagreement by consensus. If necessary, the opinion of a third reviewer was taken into consideration to resolve the disagreement.

### Data Extraction

All data were extracted independently by two reviewers (SYY and MCC) using a standard data collection form. Discrepancies were resolved through consensus and discussion with another reviewer (YJC) if necessary. The following data were recorded using a table for each eligible article: (1) name of the first author; (2) year of publication; (3) number of patients; (4) number of diagnoses of dysphagia; (5) incidence of pneumonia and number of deaths.

### Quality Assessment

The methodological quality was assessed using the Risk Of Bias In Non-randomized Studies-of Interventions (ROBINS-I) tool for the included studies. The specific domains of ROBINS-I are as follows: bias from confounding, bias from the process of participant selection, bias due to classification of interventions, bias due to deviations from intended interventions, bias from missing data, bias from measurement of outcomes, and bias from selection of the reported results. Judgments of bias were expressed as “low risk,” “high risk,” or “unclear risk.” Two reviewers (SYY and MCC) independently assessed the risk of bias in each domain. If there is any disagreement between the two reviewers, discussion was continued until consensus was achieved.

### Statistical Analysis

All statistical analyses of the pooled data were performed using RevMan 5.3 software (http://tech.cochrane.org/revman). *I*^2^ statistics were used to assess heterogeneity between studies, which measures the extent of inconsistency among the results. *I*^2^ percentages of around 25, 50, 75% represent low, medium, and high heterogeneity, respectively. Significant heterogeneity was considered to be present if *I*^2^ ≥ 50%, and the random-effects model was used for data analysis. The pooled data were considered to be homogenous if *I*^2^ < 50%, and the fixed effects model was used. The odds ratio (OR) was analyzed to evaluate differences in outcome measures (development of pneumonia and number of deaths) among patients with and without dysphagia. The 95% confidence interval (CI) was used in the analysis, and *p* < 0.05 was considered statistically significant. A funnel plot and Egger's test were also used to assess publication bias with R version 4.1.2. The funnel plot was used to determine the publication bias of individual studies based on the pooled estimate. Egger's test was used to determine whether the funnel plot was symmetrical *p* < 0.05 indicated the possibility of publication bias.

## Results

**Figure 1:**
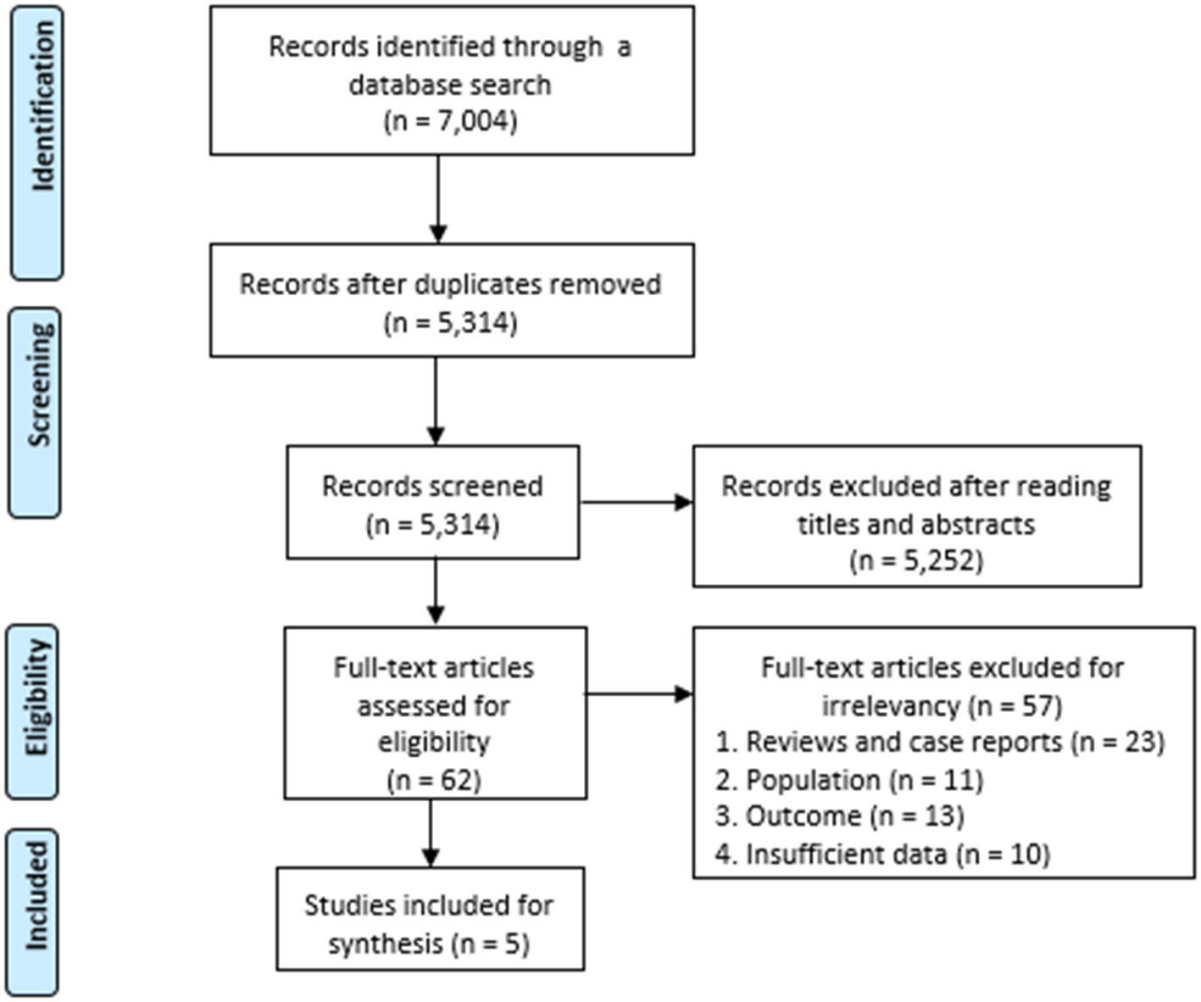
PRISMA flow diagram.

A total of 7,004 articles were identified using the search terms, and 1,690 duplicates were removed. After reading the titles and abstracts, 5,252 of the initially identified 5,314 articles that did not meet the inclusion criteria were excluded. The remaining 62 articles were assessed for eligibility, and 56 articles were excluded due to following reasons: 22 studies were reviews and case reports, 11 studies did not involve stroke patients, 13 studies did not report the prevalence of pneumonia for stroke patients with dysphagia, and 10 studies had insufficient data. Finally, five observational studies were included in this meta-analysis (Figure 1). The incidence of pneumonia and mortality rate of patients with dysphagia (intervention group) were compared with those of patients without dysphagia (control group). The characteristics of the included studies are summarized in Table 1.

**Table 1:** Characteristics of included studies.

**No**.	**References**	**Number of patients (dysphagia vs. non-dysphagia)**	**Type of stroke**	**Diagnosis of dysphagia**	**Diagnosis of pneumonia**	**Outcomes**
1	Feng et al. (8)	660 vs. 610	Stroke patients according to ICD-9 code (ischemic or hemorrhagic)	Dysphagia was diagnosed using ICD-9 787.2.	Pneumonia was diagnosed using ICD-9-CM 507 (pneumonitis due to solids and liquids).	Diagnosis of pneumonia, risk of aspiration pneumonia of stroke patients in the dysphagia and non-dysphagia groups after 1, 3, 5 years, and morality risk after 1, 3, 5 years
2	Al-Khaled et al. (18)	3,083 vs. 9,193	Clinical presentation and brain imaging (cranial CT and MRI)	Dysphagia was determined in cases of deglutition, drooling, absent swallow reflex, cough or voice change after swallowing, reduced water control, decreased oral clearance, or involuntary cough.	A combination of clinical presentations, radiologic signs detected on chest x-ray, and blood test results (C-reactive protein and leukocytes).	Pneumonia rate and mortality during hospitalization and disability defined as mRS ≥ 2 at discharge and 3 months after discharge
3	Brogan et al. (19)	312 vs. 224	Retrospective medical record review with a primary diagnosis of “Stroke” or “CVA”	Standard practice for diagnosing a patient with dysphagia was used, including clinical bedside evaluation of swallowing. Specific criteria for diagnosis were not specified.	Not specified.	Presence or absence of respiratory infection, mobility, incontinence, and dysphagia at admission
4	Walter et al. (20)	69 vs. 167	Brain imaging (cranial CT and MRI)	Clinical examination and a water swallowing test with pulse oximetry were performed. Severity of dysphagia was scored from 0 to 3.	Pneumonia was diagnosed according to the Center for Disease Control and Prevention criteria based on clinical, microbiological, and chest x-ray findings.	Presence and severity of dysphagia, pneumonia rate, and NIHSS score
5	Kwon et al. (21)	96 vs. 190	Stroke occurred within 4 days of admission; others not specified	Dysphagia was diagnosed if positive clinical signs were accompanied by pathologic findings from additional water swallowing or pharyngeal sensation tests.	Pneumonia was diagnosed if a patient had at least one of the following: (1) auscultatory respiratory crackles and fever (≥37.7°C in the axillary area); (2) radiographic evidence; (3) new purulent sputum, as mentioned in the previous report.	Pneumonia rate, hospital stay, mRS at discharge, dysphagia, and NIHSS score at admission

*AIS, acute ischemic stroke; mRS, modified Rankin Scale; NIHSS, National Institute of Health Stroke Scale*.

### Study Characteristics

In 2006, Kwon et al. (21) demonstrated that dysphagia was associated with the development of pneumonia (OR 15.56; CI 3.8–63.1; *p* < 0.001), suggesting that patients with dysphagia who aspirate were likely to have an increased risk of pneumonia. Additionally, they reported that the incidence of pneumonia was higher among patients with a higher National Institute of Health Stroke Scale (NIHSS) score (OR 3.44; CI 1.2–9.8; *p* = 0.02), possibly due to decreased consciousness or gastroesophageal reflux in the supine or recumbent position.

In 2007, Walter et al. (20) conducted a study on patients with acute ischemic stroke to investigate various risk factors that contribute to the development of stroke-associated pneumonia. In the study, they reported that dysphagia was an independent risk factor for stroke-associated pneumonia (OR 15.7; CI 5.6–43.7; *p* < 0.001). A high NIHSS score (≥10) was also associated with an increased risk of pneumonia in stroke patients (OR 2.9; CI 1.0–8.2; *p* = 0.046).

A study in 2014 by Brogan et al. (19) showed that 17% of stroke patients who had dysphagia were diagnosed with a respiratory infection. Interestingly, the study reported that dysphagia was not a significant independent predictor of respiratory infection in multivariate analysis and suggested that full assistance with mobility and incontinence were associated with respiratory infection. Respiratory infection was observed frequently in immobile and incontinent patients with an associated risk ratio of 6.5 and 3.2, respectively. The study concluded that the occurrence of aspiration pneumonia was multifactorial.

In 2016, Al-Khaled et al. (18) showed that dysphagia was independently associated with an increased risk of pneumonia (OR 3.4; 95% CI 2.8–4.2; *p* < 0.001) and case fatality (OR 2.8; 95% CI 2.1–3.7; *p* < 0.001) during hospitalization. Dysphagia was also associated with disability at discharge (OR 2.0; 95% CI 1.6–2.3; *p* < 0.001). These effects continued for 3 months after discharge; the mortality rate at 3 months after discharge was higher among patients with dysphagia compared with those without dysphagia (OR 3.2; 95% CI 2.4–4.2; *p* < 0.001), and there was a higher likelihood of disability (OR 2.3; 95% CI 1.8–3.0; *p* < 0.001).

A study by Feng et al. (8) included 1,220 patients with acute stroke (610 patients in the dysphagia group and 610 patients in the non-dysphagia group). Patients in the dysphagia group were followed for an average of 2.96 years, and patients in the non-dysphagia group were followed for an average of 3.73 years. The risk of aspiration pneumonia was significantly different between the two groups within the first year (adjusted hazard ratio (aHR) 4.69, 95% CI 2.83–7.77, *p* < 0.001), 3 years (aHR 3.49, 95% CI 2.43–5.01, *p* < 0.001), and 5 years (aHR 2.93, 95% CI 2.15–3.99, *p* < 0.001) after the stroke episode. In addition, the mortality risk was significantly different between the two groups within the first year (aHR 1.90, 95% CI 1.45–2.49, *p* < 0.001), 3 years (aHR 1.93, 95% CI 1.60–2.32, *p* < 0.001), and 5 years (aHR 1.84, 95% CI 1.57–2.16, *p* < 0.001) after the stroke episode. The study demonstrated the longitudinally relative risk of aspiration pneumonia after stroke by comparing patients with and without dysphagia.

### Risk of Bias

**Figure 2:**
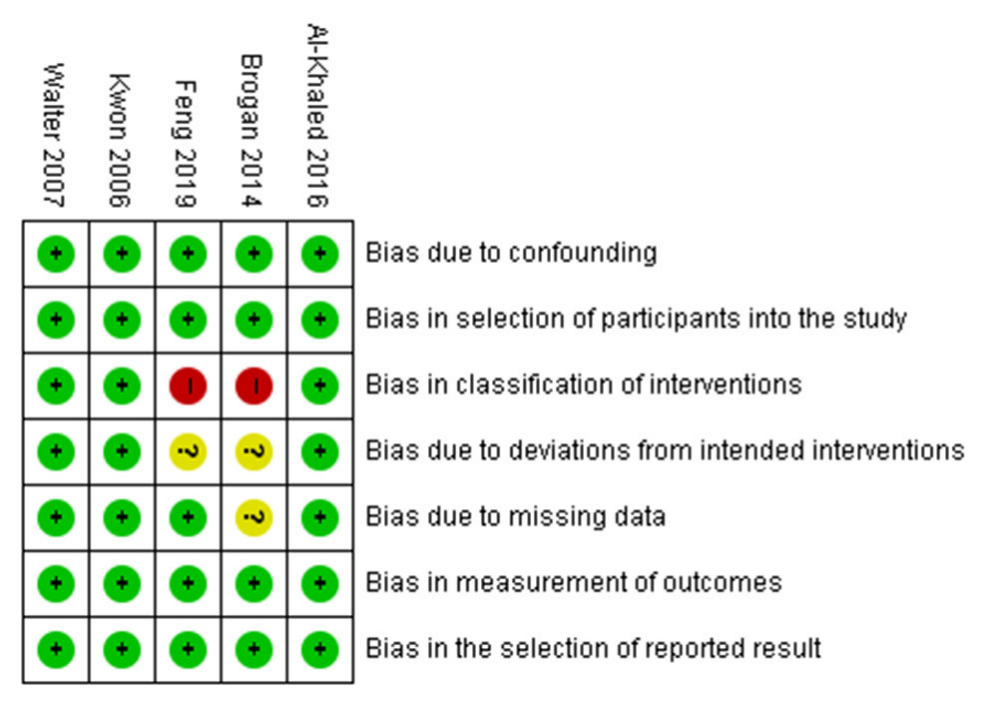
Risk of bias summary.

The risk of bias was assessed using the ROBINS-I tool for the included studies by two reviewers (SYY and MCC). All studies had a low risk of bias in the domains of confounding, participant selection, measurement of outcomes, and selection of the reported results. In the domain of classification of interventions, three studies had a low risk of bias, and two studies had a high risk of bias. In the domain of deviations from intended interventions, three studies had a low risk of bias, and two studies had an unclear risk of bias. In the domain of missing data, four studies had a low risk of bias, and one study had an unclear risk of bias (Figure 2).

### Results of Meta-Analysis

**Figure 3:**
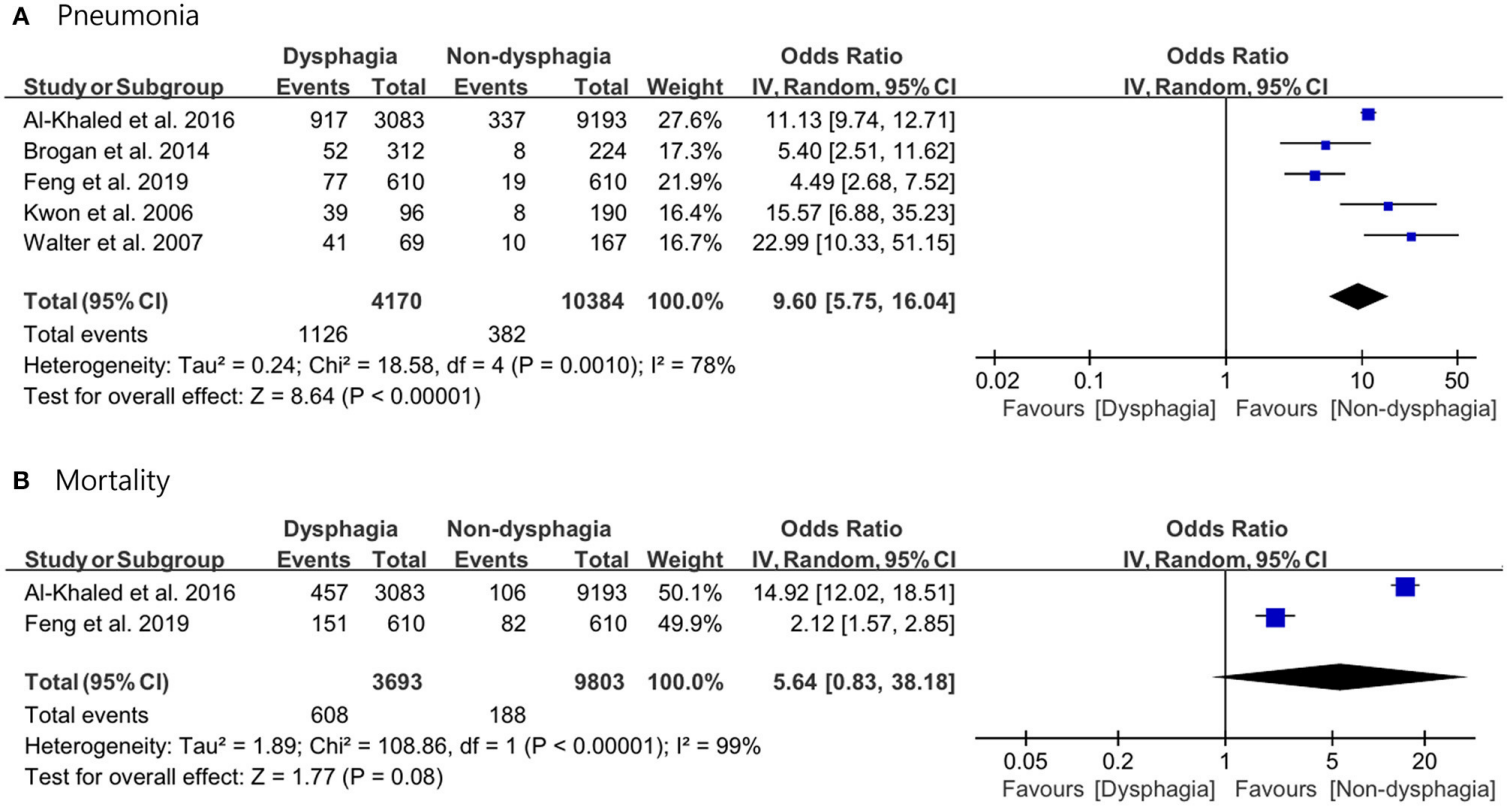
Forest plot of the incidence of pneumonia with or without post-stroke dysphagia. **(A)** Pneumonia. **(B)** Mortality.

For the pooled analysis for incidence of pneumonia and mortality rate, the random-effect model was used because the *I*^2^ values were *I*^2^ ≥ 50%. The results of the meta-analysis revealed that stroke patients in the dysphagia group had a significantly higher incidence of pneumonia compared with that of patients in the non-dysphagia group (OR 9.60; 95% CI 5.75–16.04; *p* < 0.0001; *I*^2^ = 78%). There was no significant difference in the mortality rate between the two groups (OR 5.64; 95% CI 0.83–38.18; *p* = 0.08; *I*^2^ = 99%) (Figure 3).

### Publication Bias

**Figure 4:**
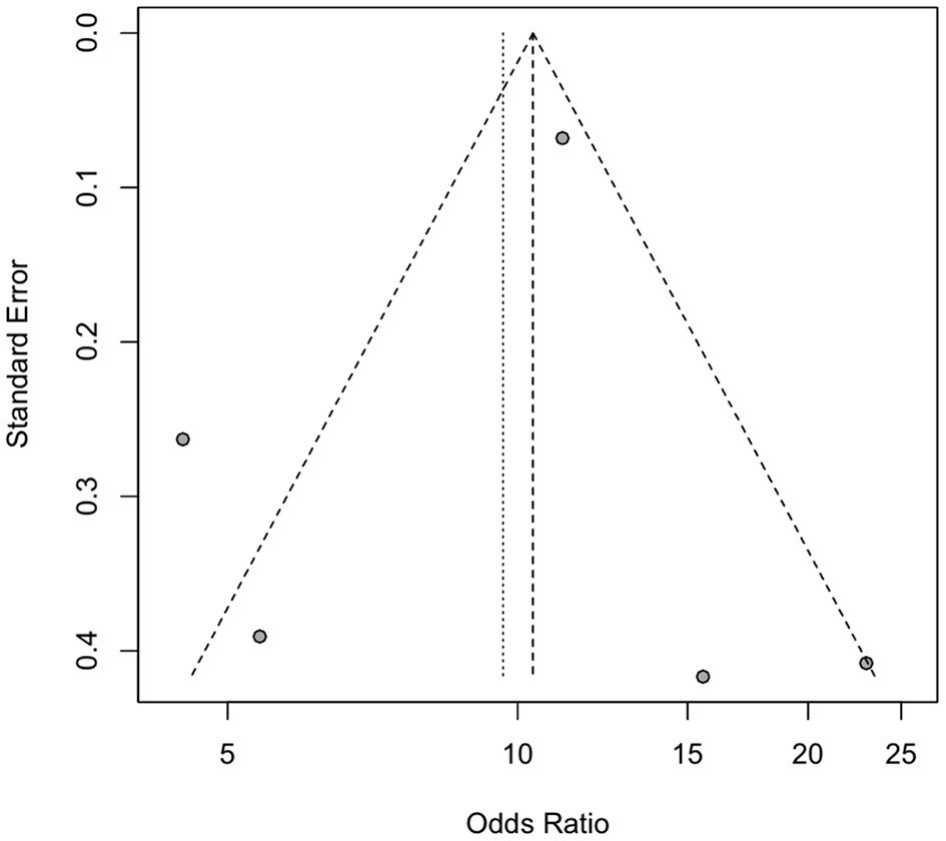
The funnel plot assesses publication bias in the included studies.

Two reviewers (SKC and MCC) evaluated publication bias using two different methods. A funnel plot and Egger's test were used to assess publication bias for studies related to pneumonia and dysphagia. As there were only two studies on mortality associated with dysphagia, the assessment of publication bias was not applicable. The funnel plot appeared to be symmetrical on visual examination, and the results of Egger's test did not reveal significant publication bias (pneumonia, *p* = 0.314) (Figure 4).

## Discussion

This meta-analysis demonstrated the close relationship between dysphagia and pneumonia, providing evidence for the increased risk of pneumonia in patients who developed dysphagia after stroke. Previous studies have reported that post-stroke dysphagia results in poor nutritional status and aspiration pneumonia, leading to prolonged hospital stay, poor functional capacity, poor prognosis, and increased mortality (8, 22). This is the first meta-analysis to demonstrate that dysphagia is significantly associated with the development of pneumonia after stroke. The results showed that the incidence of pneumonia was higher among patients with dysphagia compared with those without dysphagia. The mortality rate of patients with dysphagia was not significantly higher compared with that of patients without dysphagia after stroke.

The common signs and symptoms of dysphagia include choking on food, coughing during and after eating, drooling, effortful eating, and difficulties when swallowing (23). Post-stroke dysphagia is associated with pharyngeal muscular dysfunction and incoordination, second to central nervous system loss of control. The development of dysphagia is caused by a loss of neurological connectivity within the neural swallowing network (24). The ability to swallow may be impaired because of weakness or dysfunction of the oropharyngeal, laryngeal, and esophageal musculatures (25). Impairment in the pharyngeal phase of the swallowing process is usually associated with a risk of aspiration, leading to aspiration pneumonia. The improper movement of the bolus through the pharynx and around the larynx contributes to increased aspiration risk (26).

Although the medical, social, and psychological effects of dysphagia are significant, dysphagia is often poorly diagnosed and managed (27). The early detection and proper management of dysphagia, including adequate nutritional management and successful swallowing rehabilitation, may help prevent malnutrition and pneumonia in stroke patients (8, 11). Early pneumonia prevention is essential to reduce serious respiratory complications, such as respiratory failure, lung abscess, necrosis, sepsis, and death after stroke (8, 28).

During the acute phase of stroke, the development of pneumonia can be triggered by various factors. It has been hypothesized that stroke-induced immunodeficiency may promote bacterial infections, especially aspiration pneumonia (29). A large number of stroke patients are known to be elderly and immunocompromised (30). The complex relationship between infection and inflammatory responses may exist before and after stroke. A combination of brain-induced immunosuppression, aspiration, and dysphagia may trigger pneumonia in the acute phase after stroke (11). Dysphagia is known as a predisposing factor of aspiration pneumonia especially in elderly or patients with cognitive dysfunction (31). Impaired swallowing function in elderly is associated with physiologic decrement, including reduced tongue driving force, impaired pharyngeal constriction, and pharyngeal shortening. Patients with cognitive dysfunction also manifest dysfunctions in oral phase and pharyngeal phase (32). As most stroke patients were elderly, it may be possible that age and cognitive dysfunction may have led to susceptibility for swallowing dysfunction and post-stroke pneumonia.

Depending on the stroke severity, some patients are dependent on others for care, and some of them may be immobilized. Two studies included in this meta-analysis (20, 21). also demonstrated that a high NIHSS score, which reflects stroke severity, was associated with an increased risk of pneumonia. Decreased mobility may contribute to decreased air entry and impaired drainage of secretions from the lungs, which may result in an increased risk of pneumonia (19). In addition, it has been reported that the risk of aspiration may be high for patients with dysphagia who have a nasogastric tube, which can be associated with alterations in upper airway sensitivity, glottis injury, and laryngeal muscular dysfunction (21). These findings may explain the close relationship between dysphagia and pneumonia development after stroke.

The mortality rate of patients with dysphagia was not significantly higher compared with that of patients without dysphagia after stroke in this meta-analysis. It is possible that other risk factors of ischemic stroke death, such as hypertension, history of heart disease, consciousness disorders, hyperthermia, hyperglycemia on admission, or urinary tract infection (33), may contribute to the overall mortality rate of stroke patients.

A limitation of this meta-analysis is that the diagnostic criteria were different across studies. Diagnoses of dysphagia and aspiration pneumonia were made based on clinical symptoms and signs and neurologic and physical assessment (8, 18–21). The videofluoroscopic swallowing study is known as the gold standard assessment tool for the diagnosis of dysphagia. The actual incidence of dysphagia can be underestimated with only clinical bedside assessments of swallowing, and the reliability of diagnosis cannot be confirmed. Some studies included in this meta-analysis were performed by retrospectively reviewing medical records (8, 19); thus, the diagnosis of stroke, dysphagia, and pneumonia may not be accurate. Retrospective chart reviews rely on accurate reporting and are subject to errors in documentation (19). Therefore, patients included in these studies might not have been examined thoroughly to ensure that they had dysphagia and aspiration pneumonia. In addition, the relationship of pneumonia and dysphagia may not directly reflect the medical causality and there may be a possibility of false-positive statistical results of this meta-analysis.

## Conclusion

In conclusion, dysphagia is a significant risk factor for pneumonia after stroke. The early diagnosis and treatment of dysphagia in stroke patients are important to prevent the development of stroke-associated pneumonia. All stroke patients with clinical signs of dysphagia should be thoroughly assessed for dysphagia and should be provided with the appropriate treatment options if necessary. This study is a first step in establishing the evidence for the relationship between dysphagia and pneumonia and demonstrating the benefits of appropriate management of post-stroke dysphagia.

## Data Availability Statement

The original contributions presented in the study are included in the article/Supplementary Material, further inquiries can be directed to the corresponding author/s.

## Author Contributions

MC and SY were responsible for the study concept and design, performed the data extraction and meta-analysis, and wrote the manuscript. KS, YC, MC, and SY completed the study selection and study evaluation. YC and KS reviewed and edited the manuscript. All authors read and approved the final manuscript.

## Funding

The present study was supported by a National Research Foundation of Korea grant funded by the Korean government (grant no. NRF-2019M3E5D1A02068106).

## Conflict of Interest

The authors declare that the research was conducted in the absence of any commercial or financial relationships that could be construed as a potential conflict of interest.

## Publisher's Note

All claims expressed in this article are solely those of the authors and do not necessarily represent those of their affiliated organizations, or those of the publisher, the editors and the reviewers. Any product that may be evaluated in this article, or claim that may be made by its manufacturer, is not guaranteed or endorsed by the publisher.
