# Supplementary material for: The Relationship Between Dysphagia and Pneumonia in Acute Stroke Patients: A Systematic Review and Meta-Analysis
Source: Front Neurol. 2022 Mar 17;13:834240. doi: 10.3389/fneur.2022.834240 (PMC8970315; doi:10.3389/fneur.2022.834240)
Supplement: Supplementary file 1 [file Data_Sheet_1.docx]

**Supplementary table**

**S1. Search strategy**

A search strategy was developed to identify studies that reported the prevalence of pneumonia for stroke patients who were diagnosed with dysphagia.

The search keywords were combined as follows:

('dysphagia' OR 'swallowing' OR 'deglutition disorders' OR 'deglutition')

AND

('pneumonia aspiration' OR 'aspiration pneumonia')

**# Medical Subject Headings (MeSH) term**

| #1 | "Deglutition Disorders"[Mesh] | 53,779 |
| --- | --- | --- |
| #2 | "Deglutition Disorders"[TW] OR "Deglutition Disorder"[TW] OR "Disorders, Deglutition"[TW] OR "Swallowing Disorders"[TW] OR "Swallowing Disorder"[TW] OR "Dysphagia"[TW] OR "Oropharyngeal Dysphagia"[TW] OR "Dysphagia, Oropharyngeal"[TW] OR "aphagopraxia"[TW] OR "deglutition difficulty"[TW] OR "deglutition disorder"[TW] OR "deglutition disorders"[TW] OR "difficult deglutition"[TW] OR "difficulty in swallowing"[TW] OR "difficulty swallowing"[TW] OR "dysphagias"[TW] OR "swallowing difficult"[TW] OR "swallowing difficultness"[TW] OR "swallowing difficulty"[TW] OR "swallowing disorder"[TW] OR "swallow impairment"[TW] OR "swallowing problem"[TW] OR "Swallowing Disturbance"[TW] OR "impaired swallowing"[TW] | 40,473 |
| #3 | "Deglutition"[Mesh] | 10,034 |
| #4 | "Deglutition"[TW] OR "Deglutitions"[TW] OR "Swallowing"[TW] OR "Swallowings"[TW] OR "oropharyngeal swallow"[TW] OR "reflex, deglutition"[TW] OR "swallow (deglutition)"[TW] OR "swallow (ingestion)"[TW] OR "swallow function"[TW] OR "swallow reflex"[TW] OR "swallowing reflex"[TW] | 40,835 |
| **#5** | (("Deglutition Disorders"[Mesh]) OR ("Deglutition Disorders"[TW] OR "Deglutition Disorder"[TW] OR "Disorders, Deglutition"[TW] OR "Swallowing Disorders"[TW] OR "Swallowing Disorder"[TW] OR "Dysphagia"[TW] OR "Oropharyngeal Dysphagia"[TW] OR "Dysphagia, Oropharyngeal"[TW] OR "aphagopraxia"[TW] OR "deglutition difficulty"[TW] OR "deglutition disorder"[TW] OR "deglutition disorders"[TW] OR "difficult deglutition"[TW] OR "difficulty in swallowing"[TW] OR "difficulty swallowing"[TW] OR "dysphagias"[TW] OR "swallowing difficult"[TW] OR "swallowing difficultness"[TW] OR "swallowing difficulty"[TW] OR "swallowing disorder"[TW] OR "swallow impairment"[TW] OR "swallowing problem"[TW] OR "Swallowing Disturbance"[TW] OR "impaired swallowing"[TW])) OR (("Deglutition"[Mesh]) OR ("Deglutition"[TW] OR "Deglutitions"[TW] OR "Swallowing"[TW] OR "Swallowings"[TW] OR "oropharyngeal swallow"[TW] OR "reflex, deglutition"[TW] OR "swallow (deglutition)"[TW] OR "swallow (ingestion)"[TW] OR "swallow function"[TW] OR "swallow reflex"[TW] OR "swallowing reflex"[TW])) | **86,042** |
| #6 | "Pneumonia, Aspiration"[Mesh] | 6,082 |
| #7 | "Pneumonia, Aspiration"[TW] OR "Aspiration Pneumonias"[TW] OR "Pneumonias, Aspiration"[TW] OR "Aspiration Pneumonia"[TW] OR "aspiration pneumonia, oil"[TW] OR "aspiration pneumonitis"[TW] OR "deglutition pneumonia"[TW] | 8,842 |
| #8 | ("Pneumonia, Aspiration"[Mesh]) OR ("Pneumonia, Aspiration"[TW] OR "Aspiration Pneumonias"[TW] OR "Pneumonias, Aspiration"[TW] OR "Aspiration Pneumonia"[TW] OR "aspiration pneumonia, oil"[TW] OR "aspiration pneumonitis"[TW] OR "deglutition pneumonia"[TW]) | **9,349** |
| #9 | ((("Deglutition Disorders"[Mesh]) OR ("Deglutition Disorders"[TW] OR "Deglutition Disorder"[TW] OR "Disorders, Deglutition"[TW] OR "Swallowing Disorders"[TW] OR "Swallowing Disorder"[TW] OR "Dysphagia"[TW] OR "Oropharyngeal Dysphagia"[TW] OR "Dysphagia, Oropharyngeal"[TW] OR "aphagopraxia"[TW] OR "deglutition difficulty"[TW] OR "deglutition disorder"[TW] OR "deglutition disorders"[TW] OR "difficult deglutition"[TW] OR "difficulty in swallowing"[TW] OR "difficulty swallowing"[TW] OR "dysphagias"[TW] OR "swallowing difficult"[TW] OR "swallowing difficultness"[TW] OR "swallowing difficulty"[TW] OR "swallowing disorder"[TW] OR "swallow impairment"[TW] OR "swallowing problem"[TW] OR "Swallowing Disturbance"[TW] OR "impaired swallowing"[TW])) OR (("Deglutition"[Mesh]) OR ("Deglutition"[TW] OR "Deglutitions"[TW] OR "Swallowing"[TW] OR "Swallowings"[TW] OR "oropharyngeal swallow"[TW] OR "reflex, deglutition"[TW] OR "swallow (deglutition)"[TW] OR "swallow (ingestion)"[TW] OR "swallow function"[TW] OR "swallow reflex"[TW] OR "swallowing reflex"[TW]))) AND (("Pneumonia, Aspiration"[Mesh]) OR ("Pneumonia, Aspiration"[TW] OR "Aspiration Pneumonias"[TW] OR "Pneumonias, Aspiration"[TW] OR "Aspiration Pneumonia"[TW] OR "aspiration pneumonia, oil"[TW] OR "aspiration pneumonitis"[TW] OR "deglutition pneumonia"[TW])) | **2,424** |

**Selection of eligible studies**

Trial registers and databases including PubMed, Cochrane, Embase, and Scopus were searched for studies published up to November 20, 2021. The results of the database searches were exported to an EndNote X9 library. Duplicates were deleted using the EndNote X9 deduplication function. Two reviewers (SYY and MCC) removed irrelevant records based on the titles and abstracts. Subsequently, the reviewers examined the full text to select articles that meet the selection criteria.

Each database was searched with the following criteria:

1. MEDLINE (2,424)
   - No filters
2. Embase (4,179)
   - Publication types: article
3. Cochrane library (253)
   - trials
4. Scopus (148)
   - Document types: article

- Source types: journal

**Table S1. Eligibility criteria**

| **Population** | Studies that reported the prevalence of pneumonia for stroke patients who were diagnosed with dysphagia. |
| --- | --- |
| **Intervention** | Patients with dysphagia. |
| **Comparison** | Patients without dysphagia. |
| **Outcome** | Studies were eligible for inclusion in this review if they report on:  rate of pneumonia and mortality |
| **Study design** | Clinical trials were included in this review. |
| **Limitation** | Studies published as case reports, reviews, letters, or other undistinctive forms were excluded. Studies from all years were considered. There were no limitations on the type of screening. |
